# Antiphospholipid syndrome-associated nephropathy; a nephropathy needs classification

**Published:** 2012-01-01

**Authors:** Azar Baradaran

**Affiliations:** Department of Pathology, Isfahan University of Medical Sciences, Isfahan, Iran

**Keywords:** Antiphospholipid syndrome, Thrombotic microangiopathy, Nephropathy

**Implication for health policy/practice/research/medical education:**

A suggested classification for antiphospholipid syndrome-associated nephropathy, should be simple and practical. However, the suggestion of a new classification for antiphospholipid syndrome-associated nephropathy will involve a magnificent amount of work and will necessitate a working group, hence, more studies on this topic is suggested.

Antiphospholipid antibodies are known as a heterogeneous group of autoantibodies associated with the hypercoagulable condition affecting the vascular tree with thrombosis entitled antiphospholipid syndrome (1). Antiphospholipid syndrome is an autoimmune disorder with various etiology that contains cellular, genetic, molecular and pathogenic mechanisms. The antiphospholipid syndrome clinical characteristics are a combination of arterial and/or venous thrombosis, recurrent fetal losses, hematological events, intra-abdominal manifestations and neurological disorders. The Kidney involvement is associated with both primary and secondary antiphospholipid syndrome. Clinical features contain thrombotic microangiopathy, hypertension, renal artery stenosis, and other histological manifestations of the nephropathy, and venous kidney thrombosis (1,2). Kidney involvement is a common finding in patients with antiphospholipid syndrome. In recent years, much attention has been directed toward understanding vascular lesions in antiphospholipid syndrome-associated nephropathy. The nephropathy of antiphospholipid is characterized by thrombotic microangiopathy, fibrous intimal hyperplasia and focal cortical atrophy. It is generally believed that the nephropathy of antiphospholipid should be included in the antiphospholipid syndrome classification criteria and furthermore it seems that, investigators should define a classification for this syndrome too (1–3). However, the main question is which one of the morphologic lesions of antiphospholipid syndrome nephropathy (APS-nephropathy) should include in this classification and have prognostic significant. Indeed, vascular lesions should be categorized into acute (thrombotic microangiopathy) and chronic (fibrous intimal hyperplasia and thrombus), glomerular lesions (glomerular ballooning) and tubulointerstitial involvement (focal cortical atrophy and tubular thyroidization). In APS-nephropathy, pathologic injury is a vaso-occlusive disease which affects vessels, glomeruli and tubulointerstitial area (1–4). However, a proposed classification for APS-nephropathy, should be simple and practical. Nevertheless, the suggestion of a new classification for APS-nephropathy will involve a magnificent amount of work and will necessitate a working group, hence, more studies on this topic is suggested.

## Author’s contribution

AB was the single author of the manuscript.

## Conflict of interests

The author declared no competing interests.

## Ethical considerations

Ethical issues (including plagiarism, misconduct, data fabrication, falsification, double publication or submission, redundancy) have been completely observed by the author.

## Funding/Support

None declared.
